# Intersection of Small RNA Pathways in *Arabidopsis thaliana* Sub-Nuclear Domains

**DOI:** 10.1371/journal.pone.0065652

**Published:** 2013-06-12

**Authors:** Olga Pontes, Alexa Vitins, Thomas S. Ream, Evelyn Hong, Craig S. Pikaard, Pedro Costa-Nunes

**Affiliations:** 1 Department of Biology, University of New Mexico, Albuquerque, New Mexico, United States of America; 2 Biology Department, Washington University in St. Louis, St. Louis, Missouri, United States of America; 3 Department of Biology and Department of Molecular and Cellular Biochemistry, Indiana University, Bloomington, Indiana, United States of America; University of British Columbia, Canada

## Abstract

In *Arabidopsis thaliana,* functionally diverse small RNA (smRNA) pathways bring about decreased RNA accumulation of target genes via several different mechanisms. Cytological experiments have suggested that the processing of microRNAs (miRNAs) and heterochromatic small interfering RNAs (hc-siRNAs) occurs within a specific nuclear domain that can present Cajal Body (CB) characteristics. It is unclear whether single or multiple smRNA-related domains are found within the same CB and how specialization of the smRNA pathways is determined within this specific sub-compartment. To ascertain whether nuclear smRNA centers are spatially related, we localized key proteins required for siRNA or miRNA biogenesis by immunofluorescence analysis. The intranuclear distribution of the proteins revealed that hc-siRNA, miRNA and trans-acting siRNA (ta-siRNA) pathway proteins accumulate and colocalize within a sub-nuclear structure in the nucleolar periphery. Furthermore, colocalization of miRNA- and siRNA-pathway members with CB markers, and reduced wild-type localization patterns in CB mutants indicates that proper nuclear localization of these proteins requires CB integrity. We hypothesize that these nuclear domains could be important for RNA silencing and may partially explain the functional redundancies and interactions among components of the same protein family. The CB may be the place in the nucleus where Dicer-generated smRNA precursors are processed and assigned to a specific pathway, and where storage, recycling or assembly of RNA interference components takes place.

## Introduction

*Arabidopsis thaliana* small RNA (smRNA) silencing pathways are essential for regulating development and establishing epigenetic modifications, such as DNA and histone methylation [1], [2]. Major pathways include the microRNA (miRNA), trans-acting small interfering RNA (ta-siRNA), natural cis-antisense siRNA (nat-siRNA), and heterochromatic small interfering RNA (hc-siRNA) pathways [3], [4]. Each pathway shares a common set of steps that begin with the processing of double-stranded RNA by a DICER-LIKE (DCL) enzyme, generating 21–24 nucleotide (nt) double-stranded RNA (dsRNA) duplexes. DCL proteins interact with dsRNA-binding proteins (DRB) that aid in the dicing process [5], [6]. The smRNAs are unwound and one strand binds an ARGONAUTE (AGO) protein, which facilitates small-RNA guided target RNA cleavage, translational repression or the establishment of epigenetic modifications [7]. *A. thaliana* has four DCLs, ten AGOs, and six RNA DEPENDENT RNA POLYMERASES (RDRs) that collaborate in various permutations within different pathways. How each class of smRNA is successfully channeled through its unique pathway remains largely unknown.

Mature miRNAs are distinct from other smRNA classes by their stem-loop precursors. Those originate from Pol II-transcribed precursors that are cleaved by DCL1 to produce an miRNA, typically 21-nt in length, whose 3′-end is subsequently methylated by HEN1 (HUA ENHANCER 1). The 3′-end methylation is a unifying feature of all smRNA pathways, promoting stability of the smRNA molecule [8]. AGO1 binds the miRNA and uses the smRNA sequence to guide cleavage or translational arrest of complementary target mRNAs [1]. The miRNA pathway is essential for regulating specific transcription factors and other genes required for plant development [4].

The ta-siRNAs are produced from endogenous loci and act *in trans* to direct post-transcriptional gene silencing, being involved in developmental phase changes and organ polarity. This specific pathway is initiated upon the cleavage of non-coding primary *TAS* gene transcripts by AGO1-bound and AGO7-bound miRNA complexes [9], [10]. AGO7 binds specifically to miRNA390 and is involved in targeting *TAS3* transcripts [10]. SUPPRESSOR OF GENE SILENCING 3 (SGS3) protects the single-stranded RNA cleavage products from degradation that RDR6 utilizes as a template for double-stranded RNA production. Subsequent dicing into 21-nt ta-siRNAs occurs as a result of DCL4 activity [12], [13]. The resulting ta-siRNAs are loaded into and guide AGO1 proteins to complementary transcripts targeted for cleavage [11].

In response to biotic and abiotic stresses, transcription of two overlapping genes in reverse orientation can produce nat-siRNAs [14], [15]. For example, the opposing transcription of *P5CDH*, a stress-related gene, and *SRO5*, a gene induced by salt, results in the production of double-stranded RNAs that are processed into smRNAs [14]. In turn, these siRNAs direct the cleavage of the *P5CDH* transcripts through an unidentified AGO. In an amplification step, the cleaved *P5CDH* transcripts are stabilized by SGS3, processed into dsRNA by RDR6 and cleaved by DCL1 into 21-nt RNAs, resulting in a *cis* regulatory feedback loop to ultimately lead to salt tolerance. The nat-siRNA pathway is also dependent on RNA POLYMERASE IV (NRPDIV) [14], which is best know for its role in hc-siRNA biogenesis.

The hc-siRNA pathway is responsible for transcriptional silencing by guiding DNA methylation and changes in local chromatin structure [2], [16]. Hc-siRNAs are 24 nt in length and their biogenesis requires the activity of Pol IV [17], [18], CLASSY1 (CLSY1, a putative chromatin-remodeler) [19], RDR2, and DCL3 [3], [20]. SiRNAs bound to AGO4, coupled with RNA-directed DNA METHYLATION 1 (RDM1) and SUPPRESSOR OF TY INSERTION 5-LIKE (SPT5L) bind to transcripts produced by another plant-specific RNA polymerase, Pol V, or RNA polymerase II (Pol II), ultimately recruiting the *de novo* DNA methyltransferase DRM2 (DOMAINS REARRANGED METHYLTRANSFERASE 2) [2], [20], [21], [22], [23], [24], [25]. Pol V transcription is dependent on DRD1 (DEFECTIVE IN RNA-DIRECTED DNA METHYLATION1) [26], [27], a putative chromatin remodeler, and DEFECTIVE IN MERISTEM SILENCING 3 (DMS3), a protein similar to the Structural Maintenance of Chromosomes (SMC) [2], [22], [28].

The complexity of smRNA silencing raises important questions of how double-stranded RNA molecules can be channeled through the correct pathway. An important clue arose from studies showing that preferential binding of members of the *Arabidopsis* AGO family to smRNAs is dependent on the 5′ terminal nucleotide [29], [30]. Nevertheless, the redundancy found among DCL proteins and the contribution of RDR6 to multiple smRNA silencing pathways strongly suggests that additional factors are required for specification of a smRNA to its pathway. Interestingly, both miRNA and hc- siRNA pathway components were found to localize in nucleolus-associated structures [31], [32], [33]. Furthermore, in the case of the hc-siRNA pathway, the specific domains associated with the nucleolus are Cajal bodies (CBs).

CBs are conspicuous nuclear domains often localized to the nucleolar periphery or within the nucleoli [34], [35], [36]. These structures contain a wide variety of different proteins and small RNAs: both small nuclear RNAs (snRNAs) and small nucleolar RNAs (snoRNAs), which are factors involved in pre-mRNA splicing, pre-ribosomal RNA (pre-rRNA) processing, or histone pre-mRNA 3′ maturation; basal transcription factors for RNA polymerase I, II and III; and telomerase RNA [37], [35], [38]. It is currently thought that CBs have roles in the metabolism of different classes of small ribonucleoprotein (snRNP) particles, snoRNPs, Sm proteins, telomerase, and the U7 snRNP. In addition, CBs were found to be associated with gene-specific loci, such as histone and U2 snRNA gene clusters. It was previously shown that the hc-siRNA-processing center shares some features with CBs [32], and an analogous nuclear domain for miRNA processing has been described [31]. However, it is unknown whether all the major components of the miRNA and siRNA (ta-siRNA and hc-siRNA) pathways localize to the same or to distinct CBs at the same point in time, but it has been hypothesized that different types of CBs might co-exist [39]. Furthermore, the role of CB assembly in miRNA and siRNA component localization and biogenesis is unknown.

In this paper, we analyzed the nuclear localization and colocalization of the main components of each of the smRNA silencing pathways in *A. thaliana* to assess how these pathways are organized in the cellular compartment and whether a specific nuclear location provides a site for crosstalk among smRNA pathways. Our results suggest that a specific nuclear domain provides a site where *A. thaliana* RNAi pathways colocalize and intersect. This domain possibly constitutes a site for smRNA biogenesis, modification, effector complex assembly or storage of smRNA-related components.

## Results

### Components of the *A. thaliana* Trans-acting siRNA Pathway Localize to a Discrete Perinucleolar Structure

To evaluate where ta-siRNA pathways occur within the nucleus, we used immunofluorescence to determine the nuclear location of the proteins involved in ta-siRNA biogenesis. To avoid protein localization artifacts caused by over-expression of proteins driven by strong constitutive expression promoters, we generated *A. thaliana* transgenic lines expressing FLAG-tagged SGS3 and RDR6 under the control of their native promoters in the corresponding null mutants. Epitope-tagged proteins complement *sgs3* and *rdr6* mutant phenotypes as verified by the restoration of smRNA biogenesis and are thus functional (Figure S1). In addition to the epitope-tagged proteins, antibodies specific to native RDR6, SGS3, DCL4 or AGO7 were raised and the specificity of each antibody was determined by immunoblotting and immunolabeling in the respective mutants (Figures S2 and S3). To avoid artifacts derived from cross reaction between antibodies raised in the same organism, immunolocalizations were performed such that mouse monoclonal antibodies detected the epitope of recombinant protein in transgenic lines, whereas native proteins were detected by specific antibodies raised in rabbit.

**Figure 1:**
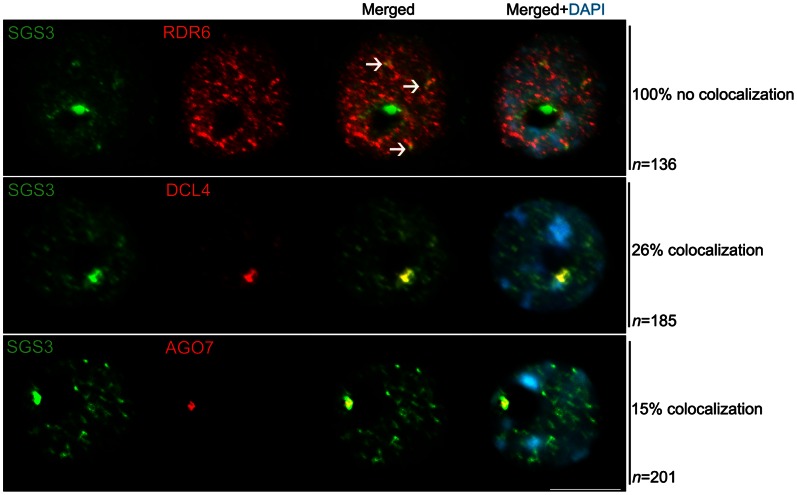
Nuclear localization of trans-acting siRNA pathway members. Double immunolocalization in interphase nuclei of RDR6, SGS3, DCL4 and AGO7 proteins indicate that, with the exception of RDR6, all the proteins are colocalized within a round-shaped signal in the nucleolar periphery (in yellow in the merged panels). In the top nuclei, RDR6 was detected with a mouse anti-Flag antibody, while SGS3 was detected with an antibody raised in rabbit against the native protein. In the middle and bottom panels, SGS3 was detected using a mouse anti-Flag antibody, whereas DCL4 and AGO7 were visualized by making use of specific native antibodies raised in rabbit. The arrow denotes colocalization foci between RDR6 and SGS3. n = number of nuclei analyzed and % indicates the percentage of nuclei with the representative immunolocalization pattern. Nuclear DNA was counterstained by DAPI (in blue). Scale bar denotes 5 µm.

As shown in Figure 1, SGS3 is uniformly distributed throughout the nucleoplasm and enriched within a prominent, round-shaped domain located in the nucleolar periphery (Figure 1). The nucleolus is the prominent nuclear compartment devoid of DNA-staining DAPI (Figure 1, in blue). Similar localization patterns were observed for DCL4 and AGO7 (Figure 1). In contrast, RDR6 does not show perinucleolar accumulation and is distributed in the nucleoplasm. The same nuclear distribution patterns of RDR6 and SGS3 were observed upon immunolocalization of epitope-tagged or the native proteins (Figure 1 and Table S1). The specificity of the antibodies recognizing the native forms of the proteins was confirmed by the absence of fluorescence signals in nuclei of the corresponding null mutant background (Figure S3).

We performed colocalization analysis to determine whether RDR6, SGS3, DCL4 and AGO7 were present at the same nuclear location. SGS3, DCL4 and AGO7 were colocalized within the perinucleolar domain in a fraction of nuclei (Figure 1). RDR6 failed to colocalize with DCL4 or AGO7 (data not shown). Only a few colocalization foci were observed between RDR6 and SGS3 in the nucleoplasm (Figure 1 top row, arrows).

### Proteins Functioning in miRNA and siRNA Pathways Colocalize within a Nuclear Region Associated with the Nucleolar Periphery

The nuclear distribution of some ta-siRNA pathway components, such as SGS3, DCL4 and AGO7, resembles the patterns previously described for key proteins involved in miRNA and hc-siRNA biogenesis [32], [20]. Two independent studies showed that both miRNA and hc-siRNA pathway proteins are enriched in a domain near the nucleolar periphery designated as a “Dicing Body” [31] or “siRNA-processing center” [20], respectively. However, it is not clear whether these nuclear domains correspond to distinct smRNA processing centers or to the same entity.

**Figure 2:**
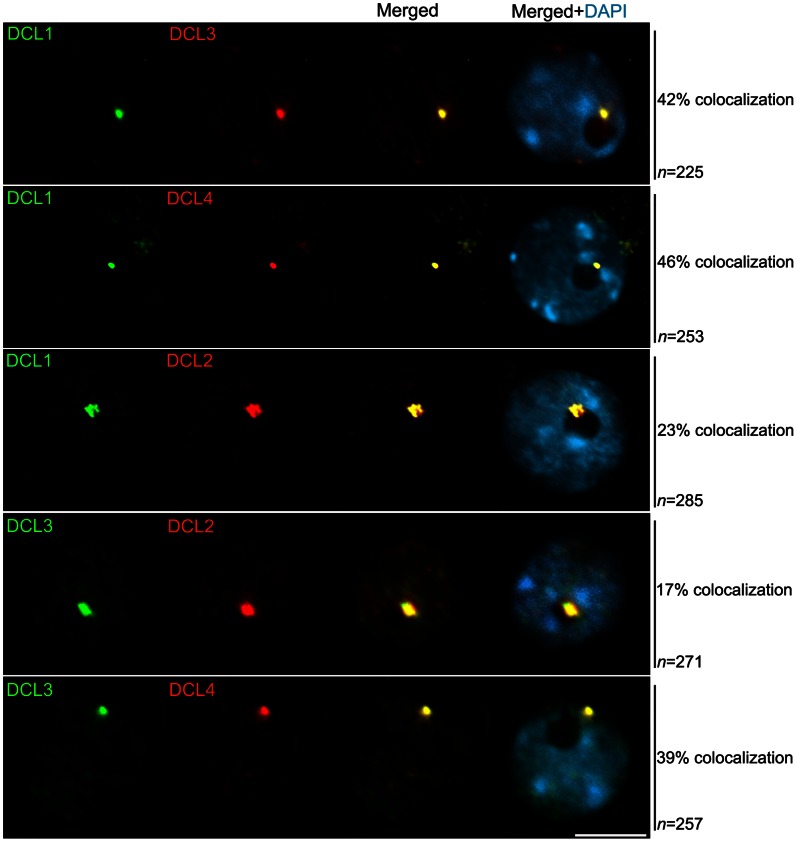
Nuclear localization of Dicer proteins. Immunofluorescence analysis of DCL1, DCL2, DCL3 and DCL4 shows that these functionally redundant proteins are colocalized within a round-shaped signal in the nuclear periphery, as suggested by the strong yellow signal displayed after merging the green and red channels. DCL proteins were detected using a specific antibody combination originated from different hosted species to avoid cross reactivity issues. n = number of nuclei analyzed and % indicates the percentage of nuclei with the representative immunolocalization pattern. Nuclear DNA was counterstained by DAPI (in blue). Scale bar denotes 5 µm.

To determine whether a unique multi-functional processing center or multiple centers exist, we colocalized all four *A. thaliana* DCL proteins, DCL1, DCL2, DCL3 and DCL4, in pairwise combinations using epitope-tagged proteins from transgenes and antibodies recognizing the native forms of the proteins. As depicted in Figure 2, DCL1, DCL3 and DCL4 colocalize near the nucleolar periphery with a frequency of 39%–46%. When colocalization was not observed, the DCL signals still occurred in the nucleolar periphery but did not colocalize, or the proteins were diffusely distributed throughout the nucleoplasm (not shown). Although DCL1 and DCL3 both colocalize with DCL2 in the round-shaped signal in the nucleolar periphery, the observed frequency was lower, between 17% and 23% (Figure 2, Fisher’s test P<0.005). This observation correlates with the high frequency of nucleoplasmic signals showed by DCL2 (67%, see Table S1).

Duplex miRNAs are methylated at the 2′ OH of its 3′ end by HEN1 [40]. HEN1 was shown to localize within a so-called “Dicing Body” which colocalizes with DCL1 [31]. To determine whether HEN1 is colocalized with other DCL enzymes, dual immunolocalization was performed between HEN1 and DCL1, DCL3 and DCL4. To localize HEN1, we produced a transgenic line bearing the protein tagged with a FLAG epitope. The complementation of the *hen1* mutation was verified by the rescue of miR167 and flower development phenotypes (Figure S4).

**Figure 3:**
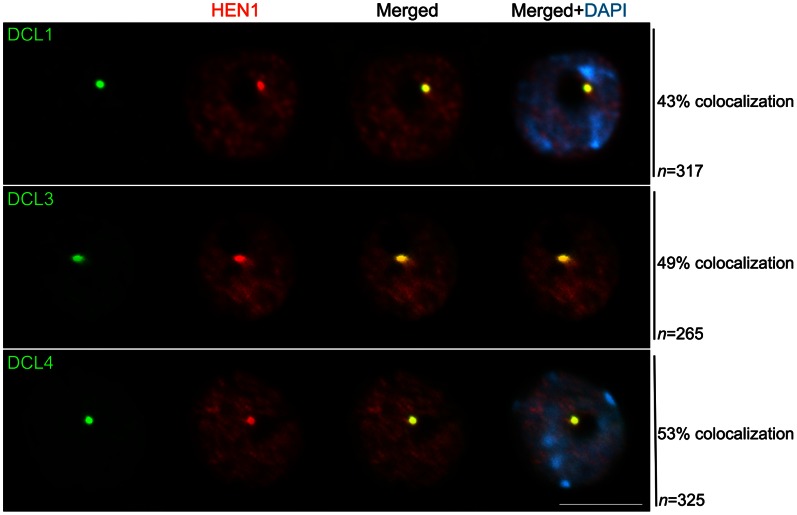
Relative localization of HEN1 and Dicer proteins. Simultaneous immunofluorescence of HEN1 and DCL1, DCL3 and DCL4 indicates these proteins colocalize within the nucleolar periphery (in yellow in the merged panels). HEN1 was visualized by immunolocalization using an anti-Flag antibody. DCL1, DCL3 and DCL4 were detected within the nucleus by using specific native antibodies. n = number of nuclei analyzed and % indicates the percentage of nuclei with the representative immunolocalization pattern. Nuclear DNA was counterstained by DAPI (in blue). Scale bar denotes 5 µm.

We found that DCL3 and DCL4 colocalized with HEN1 within a focus at the nucleolar periphery at a frequency of 43% and 53%, respectively (Figure 3). HEN1 colocalized with DCL1 at a frequency of 49% in the nucleolar periphery (Figure 3) and 21% in nucleoplasmic foci (not shown). When colocalization was not observed, HEN1 was distributed throughout the nucleoplasm while DCL proteins were found enriched in the nucleolar-associated signal or dispersed in the nucleoplasm (not shown).

**Figure 4:**
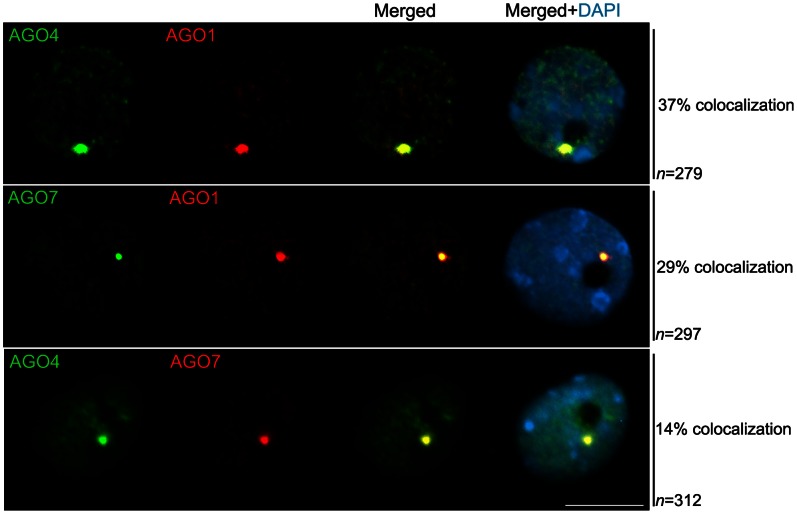
Nuclear localization of Argonaute proteins. The analysis of sub-nuclear localization of Argonaute proteins was performed by immunofluorescence. The overlay of green and red channels (resulting in the displayed yellow signal) indicates that, in a fraction of the nuclei, the proteins were colocalized within the nucleolar periphery. AGO4 was detected using an anti-cMyc antibody, whereas AGO1 and AGO7 were visualized by making use of specific native antibodies raised in chicken and rabbit, respectively. n = number of nuclei analyzed and % indicates the percentage of nuclei with the representative immunolocalization pattern. Nuclear DNA was counterstained by DAPI (in blue). Scale bar denotes 5 µm.

To test whether AGO proteins utilized by different pathways might colocalize, we performed double immunolocalization, combining epitope-tagged AGO4 detected by a mouse monoclonal antibody and antibodies specific to native AGO1 and AGO7, raised in rabbit. The specificity of AGO1 and AGO7 antibodies was confirmed by the absence of fluorescence signals in nuclei of the corresponding null mutant background (Figure S3). AGO4 and AGO1 were detected in the nucleolar domain and nucleoplasm and found to colocalize in 37% of interphase nuclei. AGO7 also colocalized with AGO1 and AGO4 but at a lower frequency, 29% and 14%, respectively (Fisher’s test P<0.005, Figure 4). AGO proteins can also be dispersed in the nucleoplasm or enriched in the nucleolar periphery without displaying colocalization (not shown).

In contrast with the results obtained with DCLs and AGOs, we observed a lack of colocalization between RDR6 and RDR2 (Figure S5), although we cannot rule out transient interactions.

### Components of the miRNA and siRNA Pathways Colocalize with Cajal Bodies

To investigate the relationship between CBs and small RNA pathway proteins, we performed systematic coimmunolocalization of all the main components of the miRNA and siRNA pathways with known CB proteins. These studies made use of antibodies recognizing specific CB components such as coilin, which molecularly defines these structures [41]; U2B’’, a spliceosomal protein which is a component of the U2 snRNP complex [42], [43]; and Y12, which recognizes methylated Arginines in Sm proteins [44]. We also developed a transgenic plant line expressing epitope Flag-tagged SmD3, a core protein of small nuclear ribonucleoprotein (snRNP) essential for splicing of primary transcripts [45], in an *smD3* loss-of-function background. Protein immunoprecipitation and western blot analysis allowed the detection of an smD3 Flag-tagged recombinant protein of the expected molecular weight, indicating that the transgenic line is functional (Figure S6).

**Figure 5:**
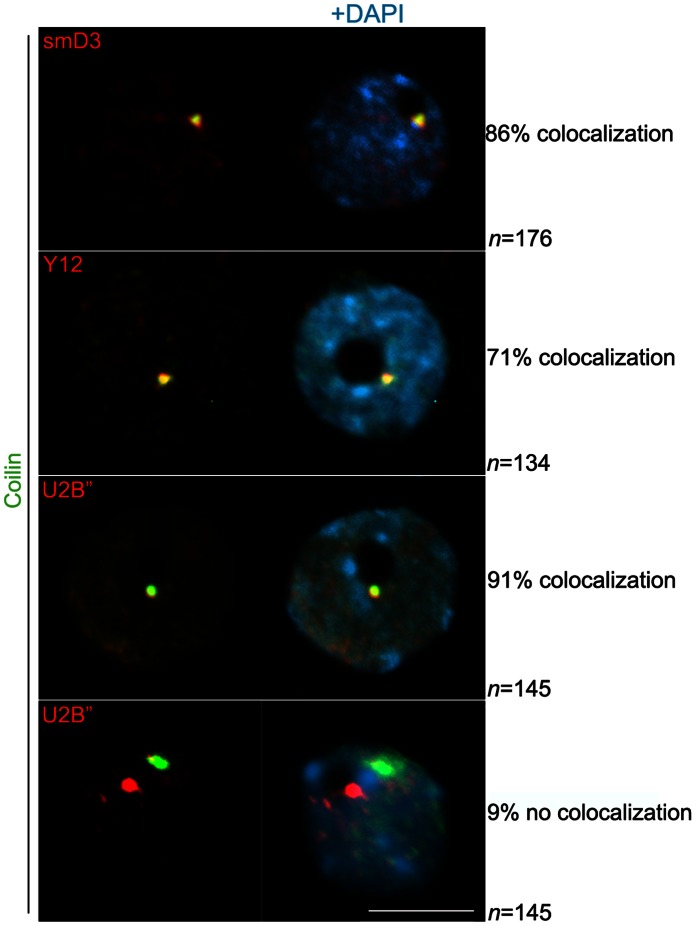
Nuclear colocalization of Cajal body components. Immunoflourescence of specific Cajal body components, U2B”, smD3 and coilin, indicates that those are colocalized in a large fraction of nuclei (in yellow in the merged panels). SmD3 was detected using an anti-Flag antibody and both coilin and U2B” were visualized by making use of specific native antibodies. Nuclear DNA was counterstained by DAPI (in blue). Scale bar denotes 5 µm.

To determine if there are multiple types of plant CB-related entities with unique protein compositions, we performed immunofluorescence to determine the degree of colocalization between the different CB markers. As depicted in Figure 5, coilin colocalized with smD3 and U2B” within a round-shaped signal located in the nucleolar periphery, in more than 86% of nuclei. Nuclear colocalization was also observed between coilin and Y12, but at a lower frequency (71%) (Fisher’s test P<0.005, Figure 5). In nuclei where CB markers were not colocalized, independent foci were observed indicating that distinct CB-like structures might coexist (Figure 5, bottom nuclei).

**Figure 6:**
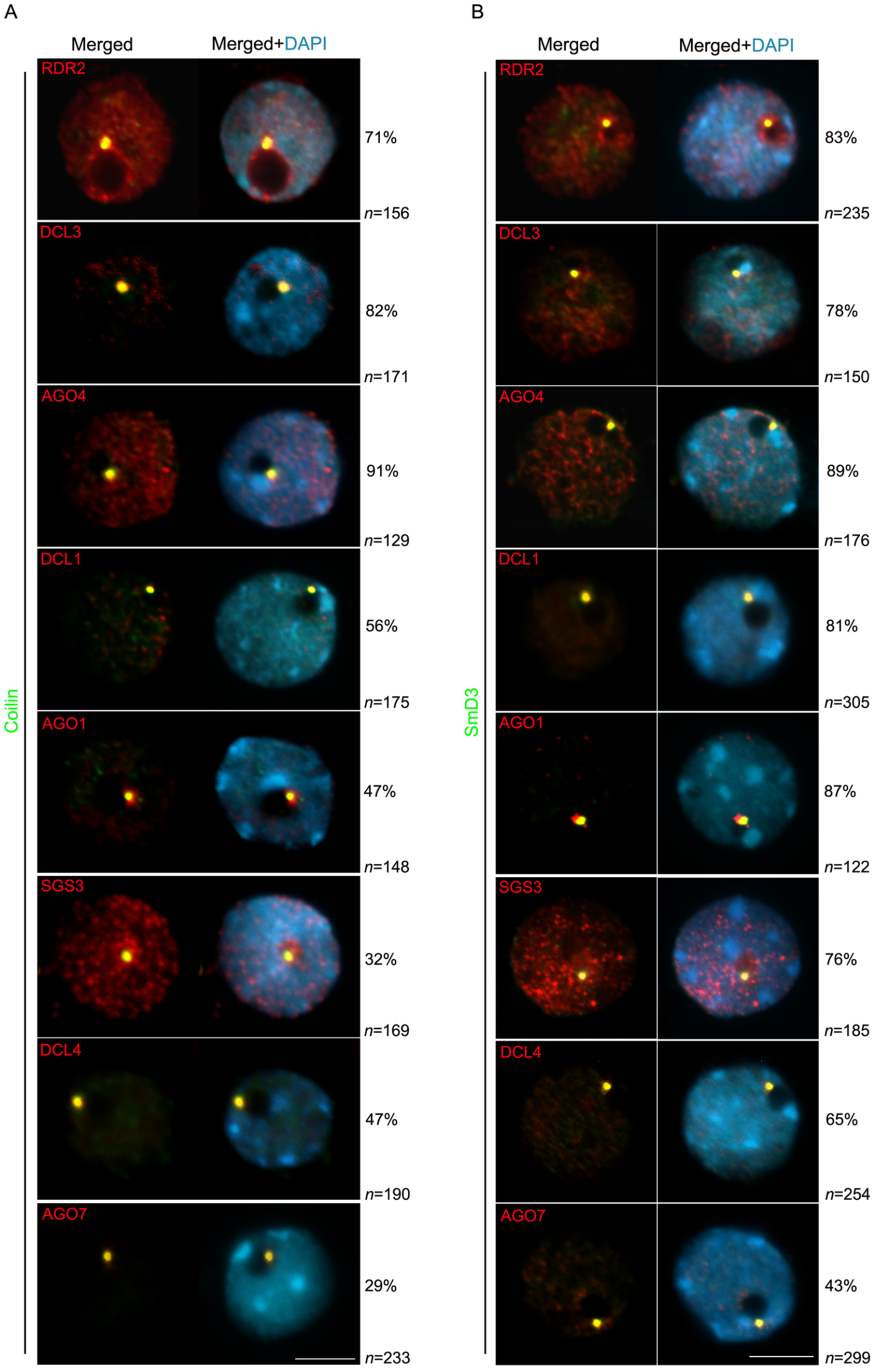
Colocalization analysis of Cajal body markers and components of miRNA and siRNA pathways. Coilin- and smD3-containing Cajal bodies overlapped with siRNA and miRNA pathway proteins in a fraction of nuclei (in yellow in the merged panels). n = number of nuclei analyzed and % indicates the percentage of nuclei with the representative immunolocalization pattern. Proteins were localized using a combination of antibodies specific to the epitope tag in the respective transgenic lines and native antibodies. Nuclear DNA was counterstained by DAPI (in blue). Scale bar denotes 5 µm.

We undertook colocalization analysis between the proteins functioning in siRNA and miRNA biogenesis with those that identify CBs to examine the possible presence of a unique or multiple siRNA-processing centers that overlap with CBs (Figure 6). All components of the miRNA and siRNA pathways colocalized with coilin and smD3 within the round-shaped signal located at the nucleolar periphery (Figure 6A and 6B). However, the frequency of colocalization differed for the two markers and among components of different pathways. Components of the hc-siRNA pathway colocalized more frequently with coilin (between 71% and 91%, Figure 6A) than those involved in miRNA and ta-siRNA biogenesis, which colocalize less frequently (between 29% and 56%, Figure 6A). Generally, smRNA pathway components were found more frequently colocalized with smD3 than with coilin, with frequencies ranging from 65% to 89% (t test, P<0.005) (Figure 6B). It is possible that spliceosomal complexes have an unknown role in RNA-mediated silencing in *A. thaliana,* similar to what has been described in fission yeast [46]. The colocalization frequencies observed with the Cajal body marker Y12 were similar to smD3 (not shown). Overall, these data indicate that the round-shaped nucleolar-associated signal, observed for the large majority of smRNA pathway components in *A. thaliana,* contains CB features.

**Figure 7:**
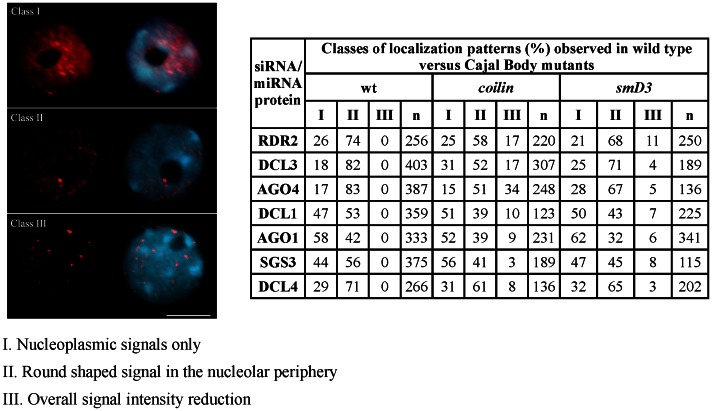
Effect of Cajal body mutants in the localization of miRNA and siRNA pathway components. Immunofluorescence of several proteins functioning in siRNA and miRNA pathways using specific native antibodies showed that the loss of function of *coilin* and *smD3* interfered with their nuclear localization. Representative nuclei for each class are displayed. In the table, n = number of nuclei analyzed and % indicates the percentage of nuclei with the representative immunolocalization pattern. Nuclear DNA was counterstained by DAPI (in blue). Scale bar denotes 5 µm.

To further investigate whether Cajal bodies are important for smRNA components’ nuclear distribution, we localized all the smRNA pathways’ components in *coilin* (47) and *smD3* loss-of-function mutant backgrounds (Figure 7). In both cases, we observed a reduction in the frequency of nuclei displaying the wild type distribution pattern of the siRNA and miRNA proteins (Class II, Figure 7 - a prominent round-shaped signal located near the nucleolar periphery) and an increased frequency of Class III nuclei (Figure 7). In particular, the enrichment in the nucleolar periphery foci was reduced upon *coilin* disruption (compare frequencies in the table of Figure 7), suggesting that these structures may be required for stability/integrity of the siRNA and miRNA pathway proteins in that nuclear domain. The interphase localization of the proteins involved in the biogenesis of hc-siRNAs was the most affected in the *coilin* background, as lower frequencies of nuclei displaying wild type patterns were observed when compared to other smRNA pathway members (compare frequency of nuclei in the table of Figure 7). These observations are in agreement with the previously suggested role of coilin-containing CBs as being required for the efficiency of RNA-dependent DNA methylation [47].

## Discussion

Overall, our analysis shows that the majority of the components that genetically define the *A. thaliana* smRNA pathways are partially colocalized within a defined region in the nucleolar periphery. The localization of DCL and AGO proteins at a common nuclear location suggests that the accumulation of smRNA precursors potentially occurs within that domain, facilitating smRNA processing and/or RISC complex assembly.

DCL enzymes participate in distinct, yet partially redundant processes, and produce miRNAs or siRNAs of characteristic sizes, ranging from 21–24 nt. This “dicing step” is generally considered a key event in generating the diversity of smRNA pathways. The colocalization of DCL proteins is a strong indication that the dicing steps of all the smRNA pathways could potentially occur in the same site in the nucleus in a significant number of cells. One possibility that may additionally account for the localization of the smRNA components in one particular nuclear domain is the requirement of 5′-RNA methylation by HEN1 in every smRNA biogenesis pathway, which could be facilitated by the physical proximity of the different players. Nevertheless, the colocalization between HEN1 and DCL proteins is consistent with the fact that these proteins act in concert with one another at the same site within the nucleus and HEN1’s requirement for proper functioning of miRNA and siRNA pathways in *A. thaliana* [48].

Upon dicing, smRNAs are loaded onto AGO proteins forming the core of RISC complexes which guide gene silencing in a sequence-specific manner. In *A. thaliana,* AGO4 mainly functions in the heterochromatic siRNA pathway, in contrast to AGO1 and AGO7, which are involved in the miRNA and ta-siRNA pathways. The observed colocalization frequencies between the three AGOs may help explain previous results suggesting that AGO proteins can bind to smRNAs with which they are not typically associated [49]. For instance, 3% of AGO4-associated smRNAs are known miRNAs that mostly associate with AGO1, and AGO4 can actively cleave targets of specific miRNAs [49]. The colocalization of AGO4 and AGO1 may help explain their partial functional redundancy.

Our data indicates that RDR6, SGS3 and AGO7 localize in the nucleus in addition to the cytoplasm [50], [51]. RDR6 is involved in multiple pathways, including PTGS of transgenes and viruses and perception and transmission of long-distance silencing signals [52], [53], hence a much broader distribution is not completely unexpected. One can hypothesize that the widespread localization of RDR6 in the nucleoplasm and the reported localization in the cytoplasm [50], [54] is a result of the involvement in multiple smRNA pathways. Furthermore, RDR6 appears to have cytoplasmic as well as nuclear functions. For instance, RDR6-dependent degradation of de-adenylated RNA occurs in the nucleus [55], and a recent report shows that dsRNAs derived from intron sequences are sufficient to trigger PTGS of the soybean FAD2-1 gene, with corresponding siRNAs detected in the nucleus [54]. In addition, RDR6 and Pol IV, a nuclear protein [20], function together in nat-siRNA biogenesis [14]. The nuclear localization of both Pol IV and RDR6 would facilitate the coordination of their activity.

We did not observe extensive colocalization between RDR6 and SGS3 within the nucleus. However, genetic experiments [12] have shown that SGS3 is required for RDR6 activity by stabilizing template RNAs, and both proteins were found at the same cytoplasmic foci, suggesting their functional dependence [50]. The absence of widespread colocalization between RDR6 and SGS3 in the nucleus may reflect transient interactions that our analysis was unable to capture or that proteins interact and colocalize mainly in the cytoplasm, as previously suggested [50]. Nevertheless, the few colocalization foci may correspond to sites of dsRNA synthesis.

**Figure 8:**
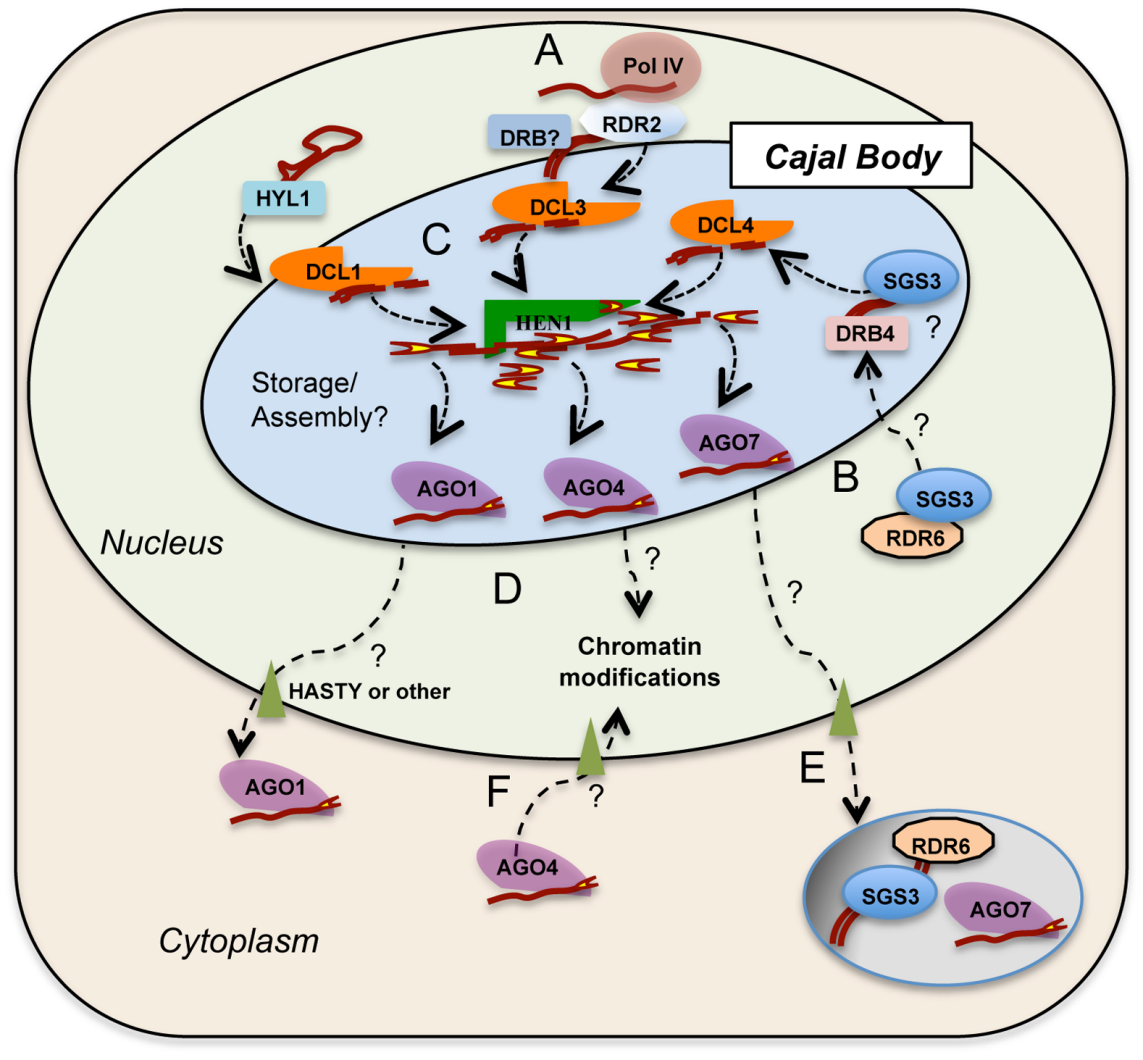
Multiple endogenous small RNA-directed silencing pathways in *A. thaliana* plants potentially intersect in a specific nuclear domain corresponding to a CB. The model depicts the scenario where pathways possibly converge in the same CB. A. RNA polymerase IV (Pol IV) transcription provides a single-stranded RNA template to RDR2. RDR2 is localized around the nucleolus and in the CB and acts exclusively in the hc-siRNA pathway. RDR2 activity produces a dsRNA precursor, processed by DCL3 into 24nt siRNA duplexes. B. SGS3 stabilization of a RDR6-derived dsRNA precursor can occur in the nucleoplasm and/or in the Cajal body. C. Dicer activity hypothetically occurs within the CB nuclear domain, with different DCLs directed to its respective substrates by DRB proteins, like HYL1 and DRB4. Upon dicing, smRNAs are methylated by HEN1 and loaded onto AGO proteins according to their size and 5′- end nucleotide. D. Loaded AGO proteins will exit the CB and be assigned to the nucleus or cytoplasm to carry out its function. Traffic to the cytoplasm might involve HASTY and other proteins not yet identified. E. In the cytoplasm, AGO7 has been found associated with RDR6 and SGS3 within a specific compartment related to post-transcriptional gene silencing [50], [51]. F. AGO4 localization to the cytoplasm as part of RISC complex maturation has been reported [57] followed by its return to the nucleus to target epigenetic modifications.

We hypothesize that the sub-nuclear compartmentalization of smRNA pathway components might play a role in their gene silencing activities (Figure 8). It is possible a stepwise process involving a cytoplasmic and a nuclear phase is required for smRNA biogenesis. Many of the smRNA components described in this study were localized in the cytoplasm in previous studies [50], [51], [56] including AGO4, which is devoted to the production of hc-siRNAs that act in chromatin [57]. Under such a model for instance, the physical proximity of DCL proteins – which seem to be exclusively localized in the nucleus [13], [54], [55], [58] – to AGOs within the same nuclear compartment could facilitate RISC complex assembly/function. DCL-produced smRNAs at the nucleolar periphery might then be exported to the cytoplasm via the exportin-5 homolog HASTY for post-transcriptional gene silencing [59], [60] and/or by action of SDE5, which is similar to a human mRNA export factor TAP. SDE5 is proposed to have a role in the transport of RNA molecules, targeting precursor or secondary RNAs to be converted into a double-stranded form by RDR6/SGS3 in the cytoplasm [61], [53]. Other possible components involved in the nucleus-cytoplasmic transport of smRNAs or their precursors could include the THO/TREX RNA trafficking complex [53], [62], [63] but its exact role in plant smRNA pathways remains unknown.

We addressed the question of whether one or more smRNA-related centers exist by testing the possible association of *A. thaliana* DCL, AGO and RDR proteins with one another and with a set of Cajal body markers. Overall, our data indicate that CBs are a point of convergence for all the different smRNA biogenesis pathways, presumably where miRNAs, ta-siRNAs and hc-siRNAs are generated or stored in close proximity (Figure 8). Cajal bodies are evolutionary conserved nuclear domains present in yeast, animal and plants cells [35], [36], [64], [65]. These conspicuous structures play major roles in a variety of RNA processing and/or ribonucleoprotein assembly processes and thereby regulate gene expression as well as assembly and transport of macromolecular complexes. There are many kinds of Cajal bodies present with a diverse array of functions. These include involvement in pre-mRNA splicing, pre-rRNA processing and modification (including methylation and pseudouridylation), telomerase assembly, viral trafficking and histone mRNA 3′ end formation [66], [67], [68], [69], [70]. A common thread to Cajal body function is the assembly of ribonucleoprotein complexes, which now possibly includes RNA Induced Silencing Complexes. If CBs are important for localization of smRNA-related proteins, we might observe defects of smRNA biogenesis in the CB mutant background. However, we did not observe any developmental phenotypes, altered siRNA accumulation or change in DNA methylation patterns in *smd3* mutant plants (Figure S7). A similar result was also previously described for *coilin* mutant lines [41], [47]. One hypothesis is that CB integrity is sufficient but not necessary for smRNA biogenesis. It is possible that CBs are involved in smRNA and/or protein processing or a yet-to-be identified function that differentially regulates the multiple endogenous siRNA pathways in *A. thaliana*. Nevertheless, as components of the smRNA machinery are not always colocalized with CBs, our data support the idea that there are multiple different types of Cajal body-related entities or smRNA-related nuclear structures and that these share different subsets of proteins. Consistent with the aforementioned hypothesis, a nuclear body, the AB-body, was described as enriched in particular RdDM components such as AGO4 and DRM2 but does not colocalize with CB markers [47].

Targeting of siRNAs and miRNAs into Cajal bodies may be an important mechanism in determining different RNA silencing outcomes. The colocalization of the miRNA and siRNA pathway components may partially explain some of the redundancy observed among pathways, which could generate a backup system in case of loss of function of any component member. The association of the RNAi-silencing machinery with CBs suggests that the latter may be a storage site, as for instance AGO7 is not functional when targeted to the nucleus [51]. Nevertheless, as miRNAs, hc-siRNAs and DCL and AGO proteins colocalize to those nuclear domains [20], [31], the possibility that the assembly of RNAi components and/or smRNA precursor processing occurs in CB cannot be ruled out.

However, the idea of smRNA processing proteins being restricted to a unique nuclear compartment may not be entirely correct, as the colocalization between proteins or with CB markers was not observed in all analyzed nuclei. In that context, smRNA pathway proteins were observed colocalizing within a round nuclear structure that does not colocalize with the CB marker or were dispersed in the nucleoplasm with variable frequencies. These observations could indicate that our results are a snapshot and the lack of complete overlapping localization patterns suggests that interactions could be transient and/or restricted to an unknown step of siRNA biogenesis. In that scenario, smRNA pathway proteins not colocalizing with the CB may correspond to a non-functional state or different steps of the corresponding smRNA processing. CBs are known to be highly dynamic in both number and size, also undergoing fusion and division [71], [72]. It is possible that the assembly of smRNA-related centers is dynamic, reflecting transient fusion/synthesis between distinct foci that are not detected by our cytological analysis. This dynamic behavior could account for the partial colocalization between and within DCL and AGO family proteins. This points to important, albeit poorly understood, functions for these nuclear sub-structures. Future work aimed at the characterization of proteins or even RNAs associated with the smRNA compartment will help to distinguish among the several functional possibilities and give further insight into the role of Cajal bodies in regulating miRNA and siRNA pathways.

## Materials and Methods

### Plant Lines

The mutant lines used in this study were: *nrpd1-3* and *nrpd2/nrpd2b* [17]; *dcl3-1* and *rdr2-1* [3]; *ago4-1* [73]; ago1-8 [74], ago7 (*zip-1*; [75]), *coilin*, SALK 148630 [47]; *hen1-1* [76]; and *smd3-1* SALK_025193. *dcl4-1*, *sgs2-1* and *sgs3*, SALK 001394 were kindly provided by Hérvé Vaucheret (INRA). All mutant lines were in a Col-0 background with the exception of *ago4-1*, *ago1-8* and *hen1-1* that were in a Ler background.

### Production of Arabidopsis Epitope-tagged Transgenic Lines

The genes were PCR-amplified from *A. thaliana* (ecotype Col-0) genomic DNA and cDNA using Platinum *Pfx* (Invitrogen) or *Pfu* Ultra (Stratgene) polymerases and primers as follows: SmD3 cDNA 5′-CACCATGAGTCGGAGTTTGGGGATTCC-3′ and 5′- CTCCTCACAGGTGGCACAGCCCCTC-3′; HEN1 primers were 5′- CACCCGCCTTACCAATCAGAGCCTTAAACC-3′ and 5′- AAGATCAGTCTTTTTCTTTTCTACATCTTCTTTCTTCCA-3′. The DCL2, RDR6 and SGS3 genomic fragments lacked a stop codon and were amplified with DCL2-F 5′-CACCGATTTGAATCTATGGAAGTTTTGGTGTTTTTA-3′ and DCL2-R 5′-GTAGTCCAGGCTGTTCTTAAGTAAGTT-3′; RDR6-F 5′- CACCTGTTCTCTGTTGCTACCTTTACTGTG -3′ and RDR6-R 5′- GAGACGCTGAGCAAGAAACTTAG-3′; and SGS3-F 5′- CACCGAAAAGGCTTTAGTTGTTGGGC-3′ and SGS3-R 5′- ATCATCTTCATTGTGAAGGCCATG -3′.

All the PCR fragments were cloned into pENTR-D TOPO (Invitrogen) and sequenced. The genes in pENTR vectors were then recombined into the pEarleyGate 302 or 202 destination vectors [77] using LR clonase and transformed into each respective mutant line by the floral dip method [78] using *A. tumefaciens* strain GV3101. RDR6 transgenic plants were generated using Agro strain LBA4404.

### Antibody Production

DCL3 and RDR2 antibodies raised in chicken were described previously [20]. DCL1 and AGO1 antibodies were raised in chicken as described in [20] against peptides CIGEPMPSVKKAKDS and MVRKRRTDAPSEGGC, respectively. Rabbit antibodies recognizing RDR6, SGS3, DCL4, and AGO7 (Sigma) were generated against peptides conjugated to keyhole limpet hemocyanin (KLH). Peptides were as follows: RDR6-MGSEGNMKKSVV; SGS3-GPMSKEKNVQGG; DCL4-RGLPQAPSKTEDR; and AGO7-KHIPSSKSRTPLLHK. Antibodies were affinity purified using peptides immobilized on SulfoLink Coupling Gel (Pierce).

### Immunofluorescence and Microscopy

Mesophyll leaf nuclei were isolated as described previously [20], [79]. Upon 4% paraformaldehyde post-fixation, the nuclei were incubated overnight at 4°C or RT with primary antibodies raised specifically for proteins involved in *A. thaliana* miRNA and siRNA pathways using a dilution of 1∶50 or 1∶100 mouse anti-Flag antibodies (Sigma). AGO4 was detected using mAb Myc (Millipore) in a dilution of 1∶200. Secondary antibodies anti-rabbit or anti-mouse or anti-chicken Alexa 594 (Invitrogen) or Alexa 488 (Invitrogen) were diluted at 1∶250 in PBS and incubated for 2 h at 37°C. DNA was counterstained with 1 µg/ml DAPI in Prolong mounting medium (Invitrogen).

Preparations were inspected with a Nikon Eclipse E800i and Deltavision (Applied Precision) epifluorescence microscopes equipped with a Photometrics Coolsnap HQ2 Mono digital camera. Images were acquired using SoftWorx software and pseudocolored and merged in Adobe Photoshop 7. Statistical analysis was performed using Fisher’s exact test and Student’s *t* test in nuclei extracted from 3–4 different plants.

## Supporting Information

Figure S1 **Mutant phenotype complementation assays of epitope-tagged lines expressing tasiRNA pathway components driven by its native promoter.** A–C. Western blot of anti-FLAG immunoprecipitated protein fractions from SGS3-FLAG (A), RDR6-FLAG (B) or DCL2-FLAG (C) transgenic lines or non-transgenic, Col-0 wt control plants. Blots were probed with anti-FLAG-HRP (1∶2000) and detected with ECL+ chemiluminescent detection reagent (GE Healthcare). The doublet band observed in the size range of SGS3 (kDa) could be due to post-translational modifications of the SGS3 protein or a cleavage event in the SGS3-FLAG protein.
(DOC)

Figure S2 **Western blot analysis of native antibody specificity.** Each antibody specifically recognizes its appropriate protein. Specificity was confirmed by the absence of band in the immunoprecipiation of wild-type tissue without the transformation of the corresponding FLAG-tagged protein. A. RDR6 protein is 137 kDa, B. SGS3 protein is 72 kDa, and C. DCL4 protein is 191 kDa.
(DOC)

Figure S3 **Native antibody labeling specificity.** Immunostaining of interphase nuclei was performed using RDR2, DCL3, AGO4, DCL1, AGO1, SGS3, DCL4 and AGO7 (all in red) native antibodies in each of the respective loss-of-function mutants. No signal was observed, indicating that the antibodies are specific for those proteins. Nuclear DNA was counterstained by DAPI (in blue). Scale bar denotes 5 µm.
(DOC)

Figure S4 **Rescue of** ***hen1*** **morphological and small RNA defects with a genomic HEN1-FLAG construct.** A. Inflorescence images of wild type Ler, *hen1-1*, and *hen1-1* transformed with the genomic HEN1-FLAG construct. Visible in the third panel is the rescue by the genomic construct of the mutant inflorescence phenotype. B. Small RNA blot probing for microRNA167 shows decreased levels in *hen1-1* when compared to wild type. Multiple lines of *hen1-1* transformed with the genomic HEN1-FLAG construct show wild type levels of microRNA167.
(DOC)

Figure S5 **Colocalization of** ***A. thaliana*** **RNA dependent RNA polymerases RDR2 and RDR6.** Immunofluorescence analysis indicates that while RDR2 (red) is localized in the nucleoplasm and at the nucleolar periphery, RDR6 (green) is mainly nucleoplasmic. RDR6 localization was performed by an anti-Flag antibody in a RDR6-epitope tagged transgenic line and RDR2 by making use of a native antibody. Interestingly, the two RDRs do not colocalize within the nucleus. “n” denotes number of nuclei analyzed and % indicates the percentage of nuclei with representative immunolocalization pattern. Nuclear DNA was counterstained by DAPI (in blue). Scale bar denotes 5 µm.
(DOC)

Figure S6 **Protein Immunoprecipitation of FLAG-tagged SmD3 recombinant protein in** ***A. thaliana smd3*** **mutant background.** SmD3-flag was immunoprecipitated from total protein extracts using anti-FLAG antibodies and detected on immunoblots using FLAG M2 antibody.
(DOC)

Figure S7 **Analysis of the** ***smd3-1*** **mutant line.** A. Two-step RT-PCR was used to evaluate knockout of *smd3*-*1* T-DNA line. Primers were designed to amplify transcription products located downstream of the T-DNA insertion but within the ORF. The absence of PCR product amplification indicates that *smd3-1* is a null allele. Genomic DNA was amplified as a control. B and C. DNA methylation levels at *AtSN1* and *5S rDNA* loci are unaffected in an *smd3-1* genomic background. *nrpd1* (*nrpd1a-3*) and *nrpd2a/nrpd2b* were used as controls. For the *AtSN1* assay, gDNA was digested with *Hae*III (CpNpN) and PCR amplified with primers specific for *AtSN1* or a control gene lacking *Hae*III restriction sites [17]. 5S rDNA methylation analysis was performed by Southern blot as previously described [17]. D. *smd3* knockout does not affect smRNA accumulation.
(DOC)

Table S1 **Interphase localization of siRNA and miRNA pathway components.**
(DOC)

Methods S1 (DOC)
