# Supplementary figures and images for: Intersection of Small RNA Pathways in Arabidopsis thaliana Sub-Nuclear Domains
Source: PLoS One. 2013 Jun 12;8(6):e65652. doi: 10.1371/journal.pone.0065652 (PMC3680462; doi:10.1371/journal.pone.0065652)

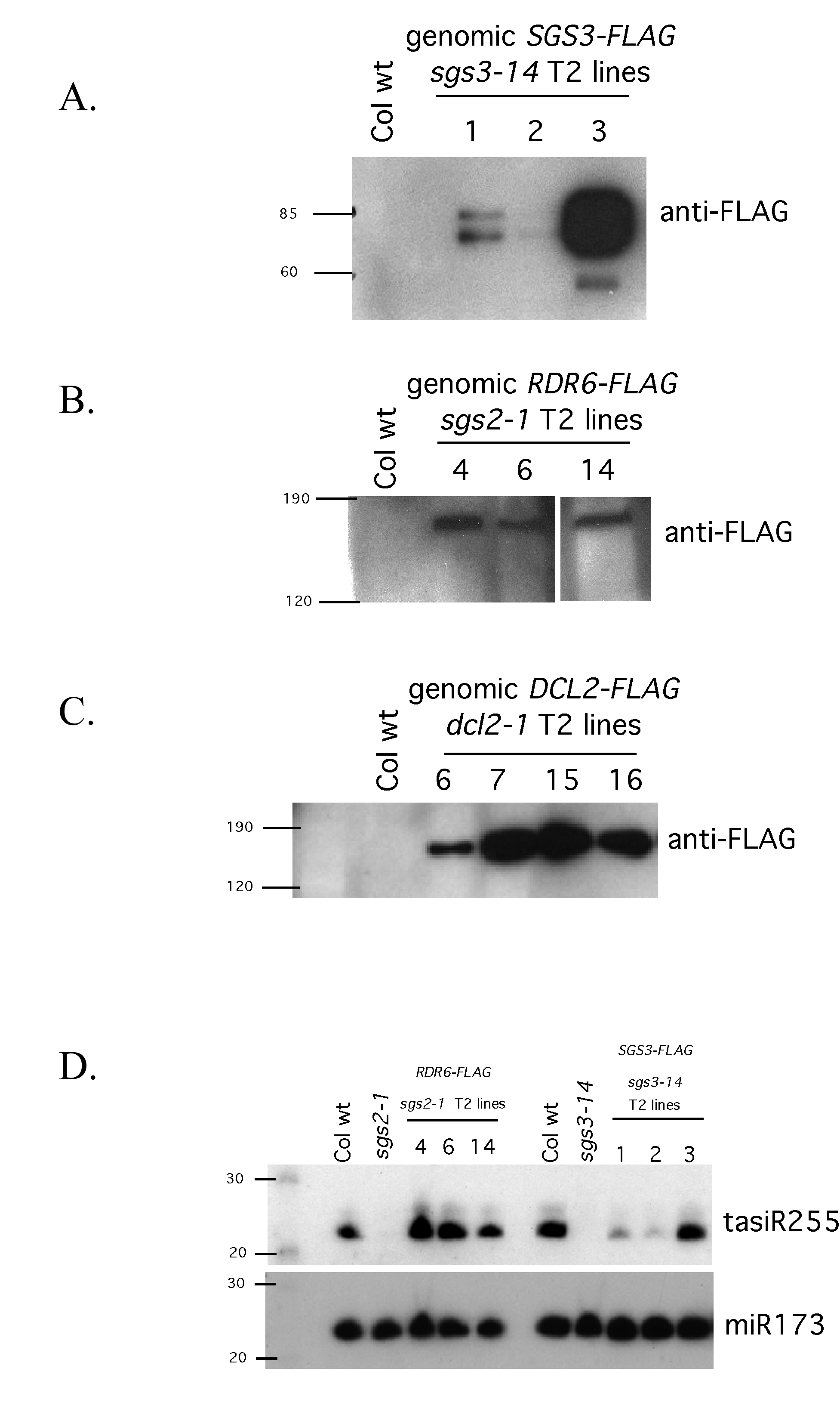

Supplement: Figure S1 — Mutant phenotype complementation assays of epitope-tagged lines expressing tasiRNA pathway components driven by its native promoter. A–C. Western blot of anti-FLAG immunoprecipitated protein fractions from SGS3-FLAG (A), RDR6-FLAG (B) or DCL2-FLAG (C) transgenic lines or non-transgenic, Col-0 wt control plants. Blots were probed with anti-FLAG-HRP (1∶2000) and detected with ECL+ chemiluminescent detection reagent (GE Healthcare). The doublet band observed in the size range of SGS3 (kDa) could be due to post-translational modifications of the SGS3 protein or a cleavage event in the SGS3-FLAG protein. (DOC) [file pone.0065652.s001.doc]

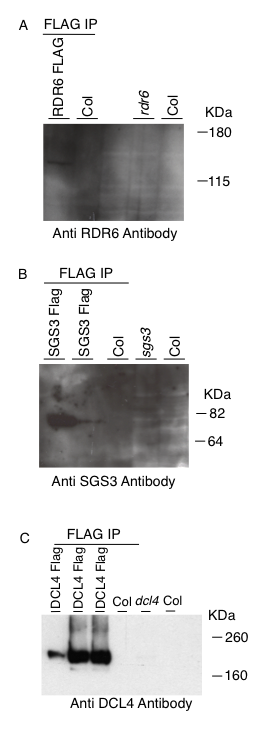

Supplement: Figure S2 — Western blot analysis of native antibody specificity. Each antibody specifically recognizes its appropriate protein. Specificity was confirmed by the absence of band in the immunoprecipiation of wild-type tissue without the transformation of the corresponding FLAG-tagged protein. A. RDR6 protein is 137 kDa, B. SGS3 protein is 72 kDa, and C. DCL4 protein is 191 kDa. (DOC) [file pone.0065652.s002.doc]

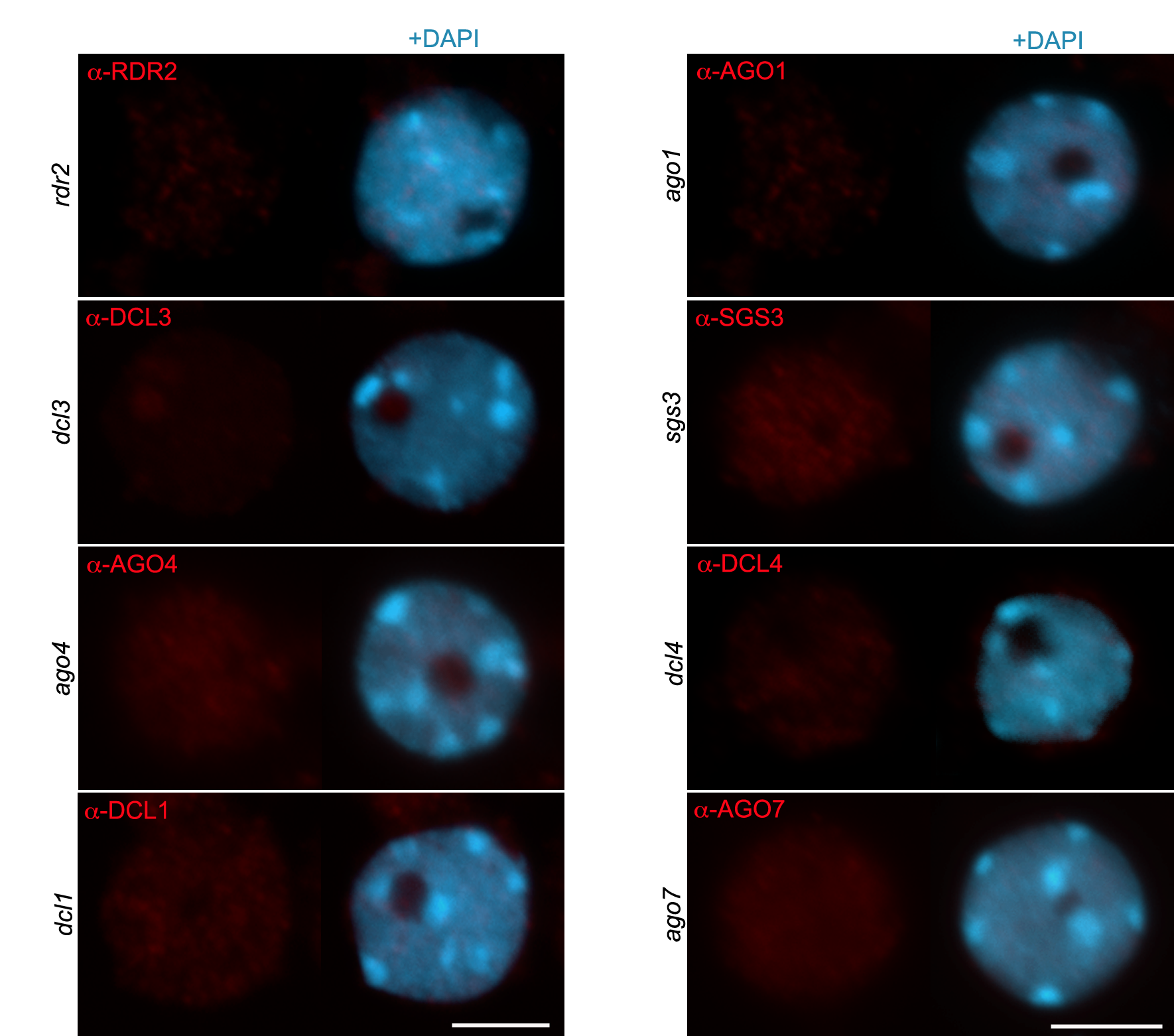

Supplement: Figure S3 — Native antibody labeling specificity. Immunostaining of interphase nuclei was performed using RDR2, DCL3, AGO4, DCL1, AGO1, SGS3, DCL4 and AGO7 (all in red) native antibodies in each of the respective loss-of-function mutants. No signal was observed, indicating that the antibodies are specific for those proteins. Nuclear DNA was counterstained by DAPI (in blue). Scale bar denotes 5 µm. (DOC) [file pone.0065652.s003.doc]

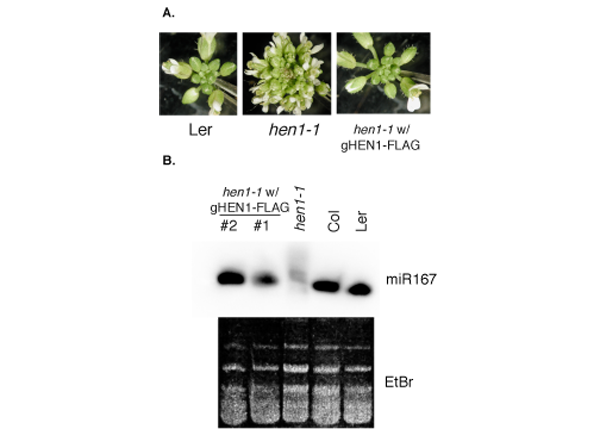

Supplement: Figure S4 — Rescue of hen1 morphological and small RNA defects with a genomic HEN1-FLAG construct. A. Inflorescence images of wild type Ler, hen1-1, and hen1-1 transformed with the genomic HEN1-FLAG construct. Visible in the third panel is the rescue by the genomic construct of the mutant inflorescence phenotype. B. Small RNA blot probing for microRNA167 shows decreased levels in hen1-1 when compared to wild type. Multiple lines of hen1-1 transformed with the genomic HEN1-FLAG construct show wild type levels of microRNA167. (DOC) [file pone.0065652.s004.doc]

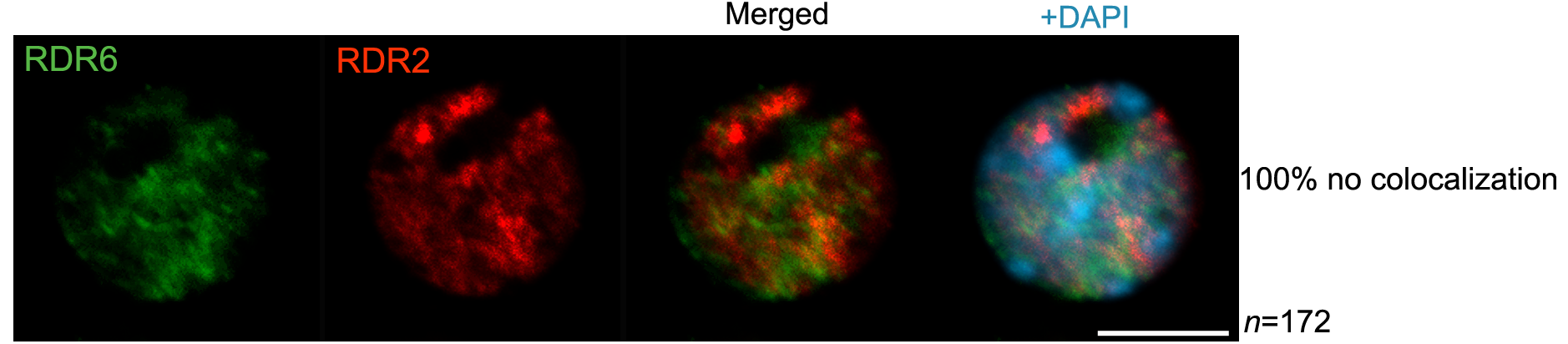

Supplement: Figure S5 — Colocalization of A. thaliana RNA dependent RNA polymerases RDR2 and RDR6. Immunofluorescence analysis indicates that while RDR2 (red) is localized in the nucleoplasm and at the nucleolar periphery, RDR6 (green) is mainly nucleoplasmic. RDR6 localization was performed by an anti-Flag antibody in a RDR6-epitope tagged transgenic line and RDR2 by making use of a native antibody. Interestingly, the two RDRs do not colocalize within the nucleus. “n” denotes number of nuclei analyzed and % indicates the percentage of nuclei with representative immunolocalization pattern. Nuclear DNA was counterstained by DAPI (in blue). Scale bar denotes 5 µm. (DOC) [file pone.0065652.s005.doc]

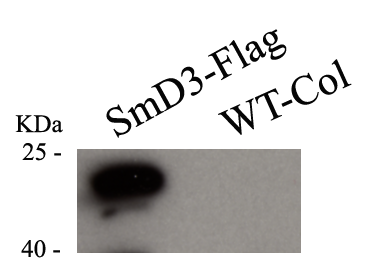

Supplement: Figure S6 — Protein Immunoprecipitation of FLAG-tagged SmD3 recombinant protein in A. thaliana smd3 mutant background. SmD3-flag was immunoprecipitated from total protein extracts using anti-FLAG antibodies and detected on immunoblots using FLAG M2 antibody. (DOC) [file pone.0065652.s006.doc]

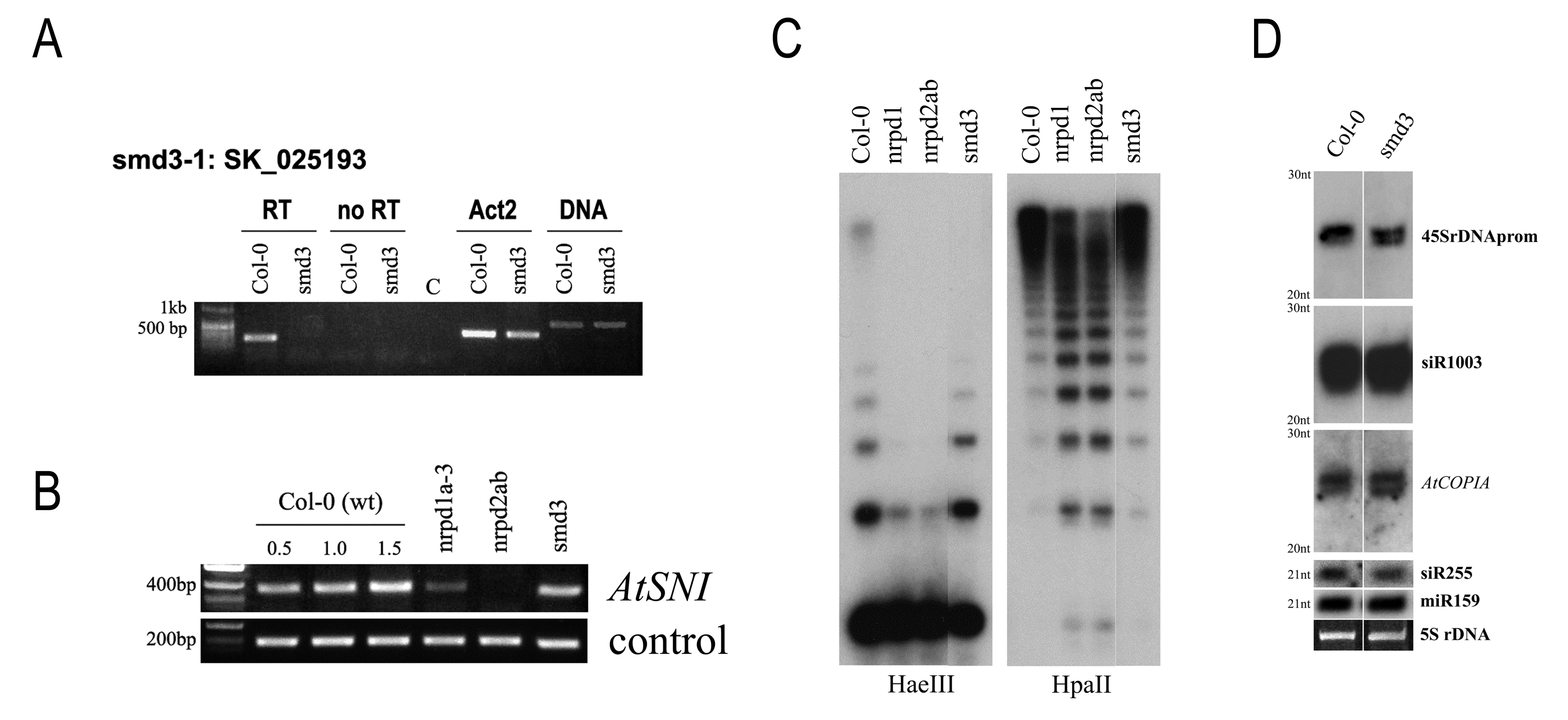

Supplement: Figure S7 — Analysis of the smd3-1 mutant line. A. Two-step RT-PCR was used to evaluate knockout of smd3-1 T-DNA line. Primers were designed to amplify transcription products located downstream of the T-DNA insertion but within the ORF. The absence of PCR product amplification indicates that smd3-1 is a null allele. Genomic DNA was amplified as a control. B and C. DNA methylation levels at AtSN1 and 5S rDNA loci are unaffected in an smd3-1 genomic background. nrpd1 (nrpd1a-3) and nrpd2a/nrpd2b were used as controls. For the AtSN1 assay, gDNA was digested with HaeIII (CpNpN) and PCR amplified with primers specific for AtSN1 or a control gene lacking HaeIII restriction sites [17]. 5S rDNA methylation analysis was performed by Southern blot as previously described [17]. D. smd3 knockout does not affect smRNA accumulation. (DOC) [file pone.0065652.s007.doc]
