# Supplementary material for: Intersection of Small RNA Pathways in Arabidopsis thaliana Sub-Nuclear Domains
Source: PLoS One. 2013 Jun 12;8(6):e65652. doi: 10.1371/journal.pone.0065652 (PMC3680462; doi:10.1371/journal.pone.0065652)
Supplement: Table S1 — Interphase localization of siRNA and miRNA pathway components. (DOC) [file pone.0065652.s008.doc]

| Protein | Classes of localization patterns observed (%) | | Total nuclei analyzed |
| --- | --- | --- | --- |
|  | I | II |  |
| RDR2 | 26 | 74 | 256 |
| RDR6 | 100 | 0 | 301 |
| SGS3 | 44 | 56 | 375 |
| HEN1 | 49 | 51 | 292 |
| DCL1 | 47 | 53 | 359 |
| DCL2 | 67 | 33 | 203 |
| DCL3 | 18 | 82 | 403 |
| DCL4 | 29 | 71 | 266 |
| AGO1 | 58 | 42 | 333 |
| AGO4 | 17 | 83 | 387 |
| AGO7 | 59 | 41 | 308 |

I. Nucleoplasmic signals only

II. Round-shaped signal in the nucleolar periphery
