# Supplementary material for: Intersection of Small RNA Pathways in Arabidopsis thaliana Sub-Nuclear Domains
Source: PLoS One. 2013 Jun 12;8(6):e65652. doi: 10.1371/journal.pone.0065652 (PMC3680462; doi:10.1371/journal.pone.0065652)
Supplement: Methods S1 — (DOC) [file pone.0065652.s009.doc]

Supplemental Methods

DNA methylation analysis

*AtSN1* methylation assays were performed on ~50ng of gDNA digested with *Hae*III. Semi-quantitative PCR conditions were 2 min at 94°C; 94°C for 30s, 53°C for 30s, and 72°C for 30s (32 cycles); 72°C for 2min. with *AtSN1* primers: 5’-ACT TAA TTA GCA CTC AAA TTA AAC AAA ATA AGT-3’ and 5’-TTT AAA CAT AAG AAG AAG TTC CTT TTT CAT CTAC-3’. A region of the *At2g19920* locus was used as control (a locus not cut by *Hae*III) and amplified with the gene specific primers 5’ CAC CCG AAC AGT TGG AAG AAG AG-3’ and 5’-GTG AGG AAC CGG TCC ATT ATT GCT-3’. PCR reactions were resolved and visualized by ethidium bromide staining following agarose gel electrophoresis (1.5% agarose; 1xTAE) [17]. Southern blot hybridizations performed as previously described [17].

Genotyping and evaluation of mRNA levels on smD3-1 SALK line

gDNA was extracted from leaf tissue and PCR amplified with Extract-N-Amp™ Plant PCR Kit (Sigma) according to the manufacturer’s instructions. SALK_025193 (*smd3-1*) was genotyped with fwd 5’-GCAAGGGAAAGAGCGCTTCACTAGG- 3’, rev 5’ TCTCTCTTCCCTCCATGTAGAG-3’ in combination with the left border primer of the TDNA insertion LBa1 5’- GCGTGGACCGCTTGCTGCAACT (SALK Institute). In order to evaluate if T-DNA insertion resulted in knock-out of the target gene, RT-PCR was used in order to determine mRNA levels in the homozygous (-/-) T-DNA lines. For identification of *SMD3* transcripts, primers fwd 5’ GCA AGG GAA AGA GCG CTT CAC TAGG- 3’ and rev 5’-TCT CTC TTC CCT CCA TGT CTT CGC G-3’ were used. Target mRNA sequence is located downstream of T-DNA insertion.

Small RNA Northern blot hybridization

Small RNAs were isolated using a mirVana kit (Ambion). Gel electrophoresis and blotting was performed as described [17]. Hybridization probes were generated using a mirVana probe construction kit (Ambion) and purified using Performa DTR Gel Filtration Cartridges, as previously described [17]. Oligos used for probe synthesis as follow: miR167, TGA AGC TGC CAG CAT GAT CTA_CCT GTC TC; siR255 (tasiRNA), TTC TAA GTC CAA CAT AGC GTA_CCTGTCTC; miR159 TTT GGA TTG AAG GGA GCT CTA_CCTGTCTC; miR173, TTC GCT TGC AGA GAG AAA TCAC; AtCOPIA TTA TTG GAA CCC GGT TAG GA_CCTGTCTC; siR1003 (5S rRNA), AGA CCG TGA GGC CAA ACT TGG CAT_CCTGTCTC; 45S rDNAprom, CAA TGT CTG TTG GTG CCA AGA GGG AAA AGG GCT ATT_CCTGTCTC.

Protein immunoprecipitation and western blot analysis

Protein immunoprecipitation and western blotting were performed as described previously [80,20] using protein extract from 3-5 g of leaf, incubated with 50 ul of anti-FLAG resin (Sigma). Blots were probed with a primary antibody against RDR6, SGS3, and DCL4, and as secondary anti-FLAG-HRP (1:2000) (Sigma) was used. The proteins were vizualized by chemiluminescence detection with ECL+ reagent (Amersham/GE).
